# A Differential Monolithically Integrated Inductive Linear Displacement Measurement Microsystem

**DOI:** 10.3390/s16030384

**Published:** 2016-03-17

**Authors:** Matija Podhraški, Janez Trontelj

**Affiliations:** 1 Letrika Lab d.o.o., Polje 15, Šempeter pri Gorici 5290, Slovenia; 2 Laboratory of Microelectronics, Faculty of Electrical Engineering, University of Ljubljana, Tržaška 25, Ljubljana 1000, Slovenia; janez.trontelj1@guest.arnes.si

**Keywords:** inductive sensor, eddy-current sensor, displacement measurement, position measurement, analog front-end, CMOS, ASIC, microtransformer, microcoil

## Abstract

An inductive linear displacement measurement microsystem realized as a monolithic Application-Specific Integrated Circuit (ASIC) is presented. The system comprises integrated microtransformers as sensing elements, and analog front-end electronics for signal processing and demodulation, both jointly fabricated in a conventional commercially available four-metal 350-nm CMOS process. The key novelty of the presented system is its full integration, straightforward fabrication, and ease of application, requiring no external light or magnetic field source. Such systems therefore have the possibility of substituting certain conventional position encoder types. The microtransformers are excited by an AC signal in MHz range. The displacement information is modulated into the AC signal by a metal grating scale placed over the microsystem, employing a differential measurement principle. Homodyne mixing is used for the demodulation of the scale displacement information, returned by the ASIC as a DC signal in two quadrature channels allowing the determination of linear position of the target scale. The microsystem design, simulations, and characterization are presented. Various system operating conditions such as frequency, phase, target scale material and distance have been experimentally evaluated. The best results have been achieved at 4 MHz, demonstrating a linear resolution of 20 µm with steel and copper scale, having respective sensitivities of 0.71 V/mm and 0.99 V/mm.

## 1. Introduction

Various types of position encoders are used in position sensing applications, most of them based on optical, inductive and magnetic sensing elements [1]. The latter rely on the effect of the Lorentz force onto the charge carriers (e.g., Hall and magnetoresistive sensors). Among these, Hall sensors are more common, since they can be fabricated using a conventional microelectronic process, allowing for a cost-efficient monolithic combination of the Hall elements and the analog sensor front-end [2] on the same silicon die, representing an Application-Specific Integrated Circuit (ASIC). Furthermore, digital signal processing circuitry can also be added to integrated sensor systems, thus creating a cost-efficient mixed signal sensor system [3]. Optical position encoders can also be integrated in a similar manner [4].

Magnetic position sensors operate by measuring the variations in spatially variable magnetic field strength. Magnetic position sensors suffer from variable offset, temperature drift, hysteresis and low immunity to noise [5]. As they require an external source of magnetic field for their operation, e.g., a magnetized scale, the availability, cost and environmental concerns about the production of rare-earth magnetic materials, may also present additional issues [6]. Since integrated optical position encoders comprise photodiodes, meaning that CMOS process modifications are required for their fabrication, the complexity and cost of optical microsensor systems are increased. Moreover, an external light source (e.g., a LED) is required [1] (p. 308). Dirt, omnipresent in industrial environments, also presents a strong issue with optical encoders [7]. However, due to their high resolution in 1 nm range [8], they are indispensable in high-precision applications.

Due to the presented disadvantages, further research of innovative integrated position encoder types is persistently encouraged by the industry, which is continuously in pursuit of dependable, accurate and inexpensive position encoders, realized in the form of an ASIC. In this paper, we present a monolithic implementation of an inductive linear incremental encoder, fabricated in using a conventional inexpensive microtechnologic process (350 nm 4-metal CMOS) without any post-processing modifications, representing a significant improvement over previously reported solutions [9,10,11,12,13] which use additional processing steps for the microinductor fabrication. We believe such inductive monolithic microsystems have the potential of substituting or supplementing conventional position encoder technologies, such as optical or Hall sensor devices.

The paper first explains the operation of the device, and presents the construction of the sensor element—an integrated microtransformer—along with its model circuit. Then, results of FEM and system-level simulations of the sensing elements’ performance with different target scales are presented, as well as the design of the measurement channel electronics supported with Cadence simulations. Finally, results of the microsystem prototype evaluation are provided with an outlook for the future work.

### 1.1. Integrated Inductive Displacement Sensors

Inductive position sensing is commonly found in large-scale devices in industrial applications, e.g., in rotary or linear variable differential transformers (RVDT/LVDT) with the position information coded in the amplitude and the phase of the output signal [14]. Integrated versions of similar devices are also reported, however with a non-monolithically integrated primary winding [10,11].

The inductive sensor output can also be observed in the frequency domain: the location of the target object can affect the inductance of a coil wired into an oscillator circuit. Integrated versions of such proximity sensors have been reported, with the coil deposited onto the surface of the silicon die during post-processing fabrication steps [12] or placed by the ASIC realized on an adjoining PCB [9].

Eddy current sensors, which are also inductive devices, commonly comprise a dual-coil structure, where the second coil voltage, induced by the current flowing through the first coil, is reduced in the presence of a conductive object due to the energy dissipation through the flow of eddy currents within the object ([1], p. 290). A dual-coil structure for eddy-current crack detection, deposited on glass substrate and employing a permalloy core, is reported by Sadler and Ahn [13]. Weiwen *et al.* reported non-integrated eddy current displacement sensors that use small planar differential coils wired into oscillatory circuits which communicate the displacement of a grating scale in frequency and phase domains [15].

Following recent developments, eddy current sensors can also be used for nanometer range measurements and have allegedly yet to reach their physical limits [16]. Wang *et al.* [17] reported of a very stable, sub-nanometer resolution displacement sensor system based on measuring the change of the inductance and resistance of a coil caused by eddy currents induced in a conductive target. Tseng and Xie [18] demonstrated a inductive system for sensing the vertical displacement of a micromirror. The system relies on adjacent transmitter and receiver coils, fabricated by the deposition of metal layers on glass substrate, operating in resonance. The two coils are coupled by the micromirror, with its displacement affecting the induced voltage of the receiver coil. The best reported resolution is 20 nm at 130 µm range.

ASICs functioning as front-ends for the conditioning and the processing of the output signals of (macroscopic) inductive sensor elements are also being researched. For example, a system comprising two ASIC frontends for an inductive proximity sensor with a frequency output is reported in [19]. An ASIC prototype for synchronous demodulation of inductive sensor output signals is reported in [20,21] by Rahal and Demosthenous. A ratiometric synchronous detection front-end for eddy current sensors employing transimpedance amplifiers is reported by Nabavi *et al.* [22,23], stating that this is the most power efficient method for interfacing eddy current position sensors.

The presented design also employs a synchronous detection demodulation method, combining the demodulation electronics with inductive sensing elements in a monolithic ASIC, which outputs a voltage signal directly related to the target displacement. The target is periodically grated and can have unlimited length, as the microsystem is essentially a prototype of an incremental linear encoder device. Thus, the presented device constitutes a bridge between larger inductive sensors and conventional integrated position sensors used in incremental encoders (e.g., Hall or optical sensors).

### 1.2. Sensor Element Operation

**Figure 1:**
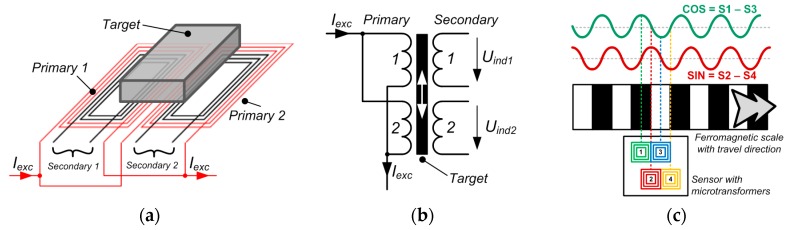
(**a**) The basic operation of the presented microsystem, consisting of two integrated microtransformers and a movable conductive and/or ferromagnetic target affecting the voltage induction between primary and secondary windings; (**b**) The schematic representation of a microtransformer pair; (**c**) The quadrature output signals are generated by moving a grating measurement scale over the microtransformer pairs. Sinusoidal signal shape is assumed.

The sensor element used in the discussed microsystem employs a concept of operation similar to a LVDT, as well as to an eddy current sensor. It is based on an integrated microtransformer pair, as presented in Figure 1a,b. The primary current *I_exc_* induces the secondary voltages *U*_*ind*1_ and *U*_*ind*2_.

The primary and the secondary winding of a microtransformer are inductively coupled by a movable conductive and/or ferromagnetic target positioned over the microtransformer. The amplitude and phase of the secondary voltage deviate with the movement of the target. If a ferromagnetic target is used, the induced secondary voltage is increased when the target is positioned over it due to flux concentration properties of the target. For a conductive (non-ferromagnetic) target, the operation is reverse [11]: when a microtransformer is completely covered with metal, its secondary voltage is reduced due to energy dissipation in the conductive target through the mechanism of eddy currents. If a conductive ferromagnetic target (*i.e.*, transformer steel) is used, the eddy current dissipation, which increases with frequency, overcomes the ferromagnetic effect at a specific margin frequency [11,17]. At this frequency, which was found using FEM simulations to be typically in the range between 1–10 MHz and is strongly dependent on the material conductivity and permeability [24], the effects of eddy currents and the magnetic reluctance of the ferromagnetic material, may cancel each other, resulting in a very low sensitivity of microtransformers operating around that frequency [17].

If the target is made periodic, *i.e.*, to consist of exchanging full and void areas with an equal width, and is moved over a microtransformer pair, incremental outputs are obtained, as illustrated using an example of a ferromagnetic scale in Figure 1c. Conductive scales can also be used. The areas should always cover a single microtransformer to maximize the effect of the target on the secondary voltage.

When the full half-period of the ferromagnetic scale is positioned centrally over the first microtransformer, the coupling between the primary and the secondary winding is strongest for this microtransformer. Conversely, the coupling is weakest for the third microtransformer as the void half-period is positioned over it. In this situation, the voltage difference of the microtransformer pair is maximal. In the same position, the second and fourth microtransformer have equal outputs as they are set to cover equal areas of full and void half-periods. Therefore, their differential signal is zero. The outputs change periodically if the target moves, returning the sine and the cosine differential quadrature signals given by the equations in Figure 1c. Again, the signals would be inverted for a conductive non-ferromagnetic scale.

Incremental position encoders (e.g., those based on optical [4] and Hall Effect [25] devices) generally utilize quadrature signals. If they have a sinusoidal shape, the arctangent of their amplitude ratio:

(1) $$x=\text{arctan}\left(\frac{\sin\frac{2\pi }{P}x}{\text{cos}\frac{2\pi }{P}x}\right)$$

returns a periodically linear positon *x* over a half of a scale period *P* [25].

The implementation of the integrated differential transformer as presented in Figure 1 differs from the previously known designs [10,11] in the fact that it is completely fabricated in a CMOS process, and in that it utilizes a single excitation coil and a counter-phase connection of the secondary windings, which are both features of a generic LVDT [14] (p. 85). The presented structure, employing two primary windings and an amplifier for the subtraction of the secondary signals, introduces more symmetry into the IC layout, thus reducing the undesirable common mode voltage in the differential signal. This common mode voltage can be due to noise, electromagnetic interference (EMI) and, most importantly, the capacitive coupling between the primary and secondary winding.

## 2. Microsystem Design and Simulation

**Figure 2:**
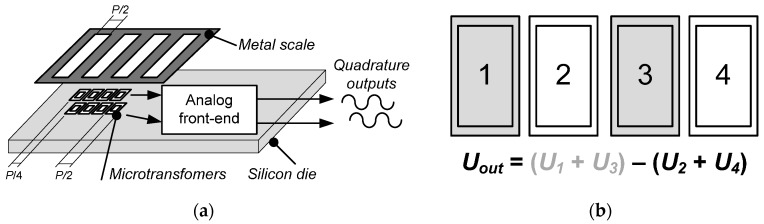
(**a**) A block representation of the presented microsystem with a metal scale of period and quadrature output signals; (**b**) The summation scheme of the presented microsystem comprising four microcoils per channel.

The design of the prototype microsystem with a piece of a target scale is presented in Figure 2a. It consists of a silicon die comprising the microtransformers along with analog front-end electronics for signal conditioning and processing. The external dimensions of the primary and the secondary microcoil of the microtransformer are 750 by 495 µm and 576 by 321 µm, respectively, with the scale period *P* of 1 mm. The center-to-center distance of two adjacent microtransformers equals *P*/2, *i.e.*, 500 µm. As the microsystem is intended to find its final application in an incremental linear encoder, it is desired that its outputs have a round period, facilitating the interpolation and the evaluation of the signal. Furthermore, the period size and consecutively the microtransformer dimensions were also directed by the limited silicon area for prototyping the ASIC.

The number of microtransformers can be increased to improve the output signal level by summing the output voltages of coils with the same position inside a distinct scale period. The summation scheme for the discussed sensor system prototype comprising four microtransformers per channel is presented in Figure 2b. As presented in Figure 2a, the sensor is comprised of two channels shifted for a quarter of the scale period, thus yielding quadrature output signals.

### 2.1. Microtransformer Design and Its Model Circuit

**Figure 3:**
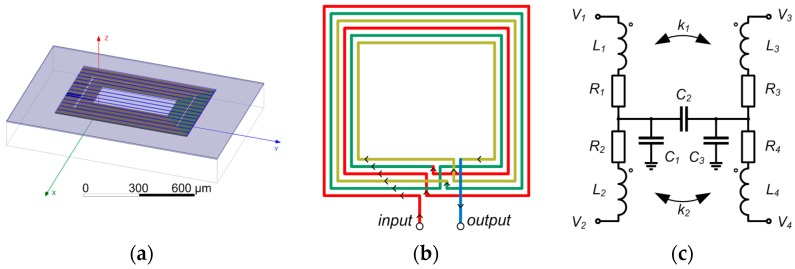
(**a**) The 3D model of a single microtransformer introduced into the parasitic extraction software; (**b**) The winding structure used in the microtransformers. Layer thicknesses and color coding are given in Figure 4a; (**c**) The model circuit of a single microtransformer. The components’ values are given in Table 1.

**Figure 4:**
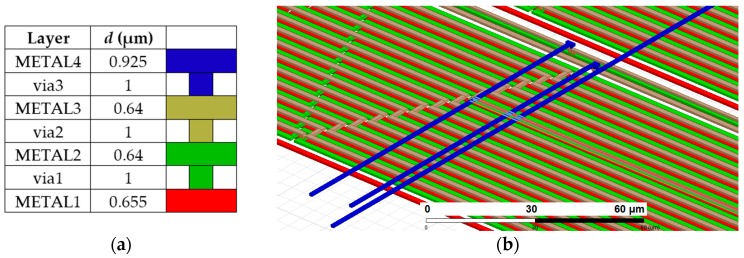
(**a**) Thicknesses (*d*) of metal layers used for the microtransformer construction; (**b**) A three-dimensional representation of the microtransformer windings. The same layer coloring is used in both figures.

A 3D model of the microtransformer is presented in Figure 3a and Figure 4b. Both the primary and the secondary winding comprise 45 windings in three metal layers (15 per layer) with the bridging interconnections realized in the fourth metal layer. A standard 350 nm microtechnologic process is used. The winding trace width is 1.5 µm and the spacing between the traces is 0.2 µm. The structure of the layered winding is presented in Figure 3b. Only one winding (primary) is shown with two turns per layer to clearly illustrate the principle. The current flow direction is indicated with arrows. The secondary winding with the same structure is placed concentrically in the middle of the primary winding. The inter-winding capacitance is reduced by placing neighboring windings in different heights. This is believed to bring an approximate 60% reduction of the inter-winding capacitance (simulated using FEM as the ratio between the capacitance of two parallel rectangular conductors with trace dimensions, placed in the same and in another metal layer).The trace width was chosen according to process specification of recommended maximum current per metal trace width (1 mA/µm), to allow the nominal excitation current of 1 mA. The trace was widened to 1.5 µm as a safety measure to prevent the heating of the ASIC, which would affect the operation of the electronics. A rectangular coil shape was selected to maximize the silicon space utilization. The turn number was mainly directed by the target primary resistance range of 5 kΩ: the excitation voltage amplitude limit is limited to 5 V, which gives a nominal current of 1 mA at that resistance. This voltage limit is equal to the ASIC supply voltage and has to be followed due to the requirements of the IC ESD protection structures. The turn count also governs the windings’ inductance and their coupling factor *k*, which is maximized by positioning the windings concentrically.

A model circuit of a single microtransformer (without the target) is presented in Figure 3c. Connection Terminals 1 and 2 are used for the introduction of the primary (excitation) current into the system. The induced voltage is measured at Terminal 3 of the microtransformer. Terminal 4 is used for the reference potential connection, *i.e.*, the DC bias voltage for the amplifier.

ANSYS Electronic Desktop (Q3D Extractor) software package was used to determine the model circuit parameters. A GDS file, including the metal layers from the IC layout database, was imported into the software. The model circuit extraction process is not extensively time- and memory-consuming: it took approximately 45 min on 16 cores of a Xeon E5-2680 processor, requiring less than 2 GB of RAM. The extraction results are given in Table 1.

The model circuit component values indicate high winding resistances (e.g., 5314 Ω for the primary). This is due to the small cross-sectional area of the trace and the large number of windings. The total primary load resistance of all eight microtransformers (wired in parallel in the IC) is therefore 664 Ω.

The capacitances are relatively large, causing a significant capacitive coupling between the primary and the secondary winding. However, this effect is effectively mitigated by the symmetry of the coils forming a pair and the differential operation of the sensor: since the capacitively transferred signal is equal for all microtransformers, it is effectively subtracted out of the differential signal. Also, the ASIC substrate ground must be well-defined to suppress the coupling between the windings through the substrate.

**Figure 5:**
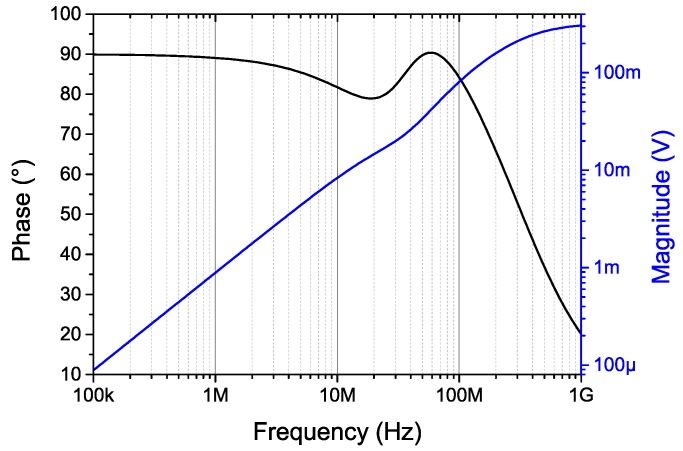
The voltage transfer function of the microtransformer as presented in Figure 3. The circuit is excited into the node *V*_1_ with an AC source of unity magnitude, and the output is measured in node *V*_3_. Nodes *V*_2_ and *V*_4_ are grounded.

The simulated transfer function of the microtransformer is presented in Figure 5. Its bandwidth (over 100 MHz) does not limit the system operation, which is instead limited by the analog front-end electronics to approximately 10 MHz.

High winding resistances result in strongly damped resonant circuits formed by the microtransformers’ inductances and parasitic capacitances. The approximate quality factor *Q* of the primary winding is 0.30 with a resonant frequency of 111 MHz. For the secondary winding, the quality is 0.42 and the resonant frequency is 183 MHz. These resonances are difficult to discern from Figure 5 due to strong resistive damping.

The use of such in-chip high-resistance and high-inductance microcoils with thin conductor widths in the resonance is limited due to their low *Q* and high resonant frequency. Therefore, the presented microsystem relies on non-resonant inductive coupling. We found the microtransformer parameters satisfactory for the task.

However it would be interesting to investigate the CMOS-technology feasibility and performance of microtransformers using wider traces, similar to the displacement-sensing inductor reported in [18] with a significantly larger size (2 by 2 mm), a quality factor of 14 and a lower resonance frequency (9.4 MHz).

### 2.2. Simulations of the Target Effect

The microtransformer transfer function presented in Figure 5, indicates an output magnitude range of approximately 1–10 mV in the frequency range of 1–10 MHz if 1 V excitation is used. However, a full 3-D simulation needs to be carried out to determine the modulation parameters of the output signal if a target scale is moved over the microtransformers. COMSOL Multiphysics FEM simulation software has been used for the task.

**Figure 6:**
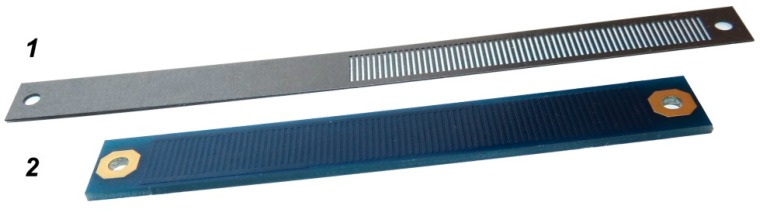
Measurement scales used for the simulation and the characterization of the microsystem. 1—transformer steel laser cut scale with 0.35 mm thickness; 2—PCB scale with 35 µm copper thickness. Full and void parts of the scale are each 0.5 mm wide, resulting in a scale period of 1 mm.

The performance of a microtransformer pair (as presented in Figure 1a) has been evaluated for a copper scale fabricated as a printed circuit board (PCB), and a laser-cut ferromagnetic scale, made from transformer steel (Acroni M330-35A [26]). The simulated target scales were designed to represent the actual scales used for the microsystem characterization, presented in Figure 6.

**Figure 7:**
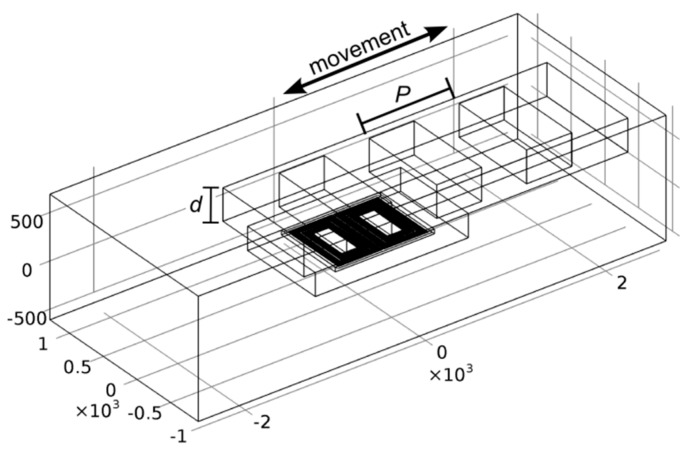
The simulated microtransformer pair geometry with a scale period *P* of 1 mm and a *d* = 0.35 mm thick target scale, representing the ferromagnetic scale type. The direction of the scale movement is indicated by an arrow. Dimensions are given in µm.

In the simulator, the conductivity of copper was set to 5.9 × 10^7^ S/m, while the conductivity of steel was set to 2.2 × 10^6^ S/m according to [27] (p. 14/3). The relative magnetic permeability of the steel was set to a generic value of 10 according to [28], since we have been unable to obtain exact electromagnetic properties for the used scale material. The microtransformer was modeled as a planar rectangular spiral coil. A 3-D image of the simulated structure is presented in Figure 7.

Since the cross-section of the microtransformer aluminum conductors (0.64 by 1.5 µm) is small in comparison to the dimensions of the target and to the skin depth of aluminum (37 µm at 5 MHz [29], p. 315), which assures an uniform current distribution in the conductor cross-section), the conductors can be modeled as current-carrying lines, significantly reducing the complexity of the FEM mesh. Despite this simplification, a displacement sweep with the target position calculated in 30 steps of a 1 mm displacement range still takes several hours for a single frequency, requiring more than 30 GB of RAM in a workstation using 16 cores of an Intel Xeon E5-2680 processor. Due to the complexity of the simulation we have chosen to simulate only a single pair of microtransformers instead of two prototyped pairs.

The primary current amplitude was set to 1 mA, which approximately corresponds to the excitation voltage amplitude of 5 V at 5 kΩ primary winding resistance. The simulation was carried out in the complex domain. The vertical distance between the target and the microtransformers was 200 µm. The secondary induced voltage can be determined using line integration of the vector magnetic potential ***A*** over a closed loop *L* of the secondary winding ([29], p. 192 and 197):

(2) $$U_{ind}=-2\pi f\oint_{L}^{□}A\cdot dl$$

**Figure 8:**
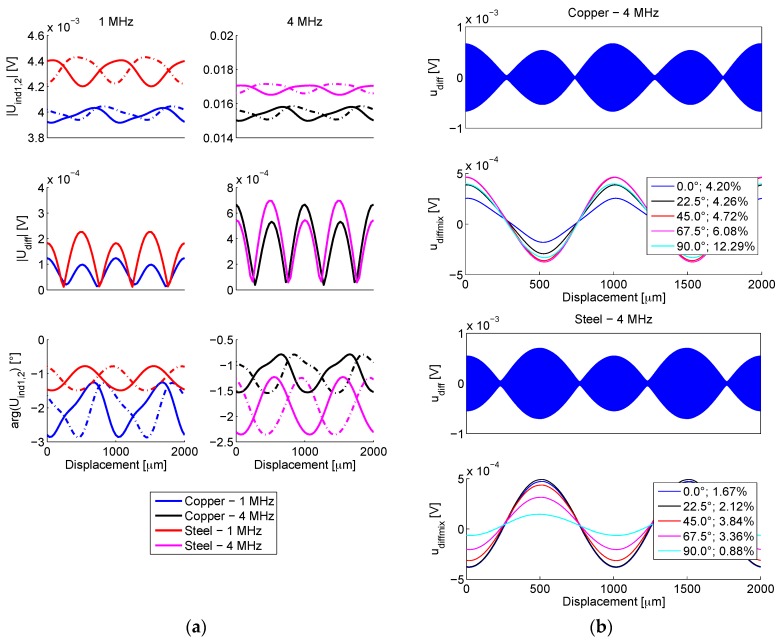
(**a**) Simulated displacement dependence of the amplitude and phase of the secondary induced signals *U*_*ind*1_, *U*_*ind*2_ (shown in dotted lines), and the differential signal *U_diff_*; (**b**) The time-domain differential signal *u_diff_* for the steel and copper scale at 4 MHz is shown. A percent THD value of thesynchronously demodulated signals *u_diffmix_* at their corresponding phases of the mixing signal ϕ_*mix*_ is also given in the legend.

For each microtransformer, an array of complex induced voltages is returned, corresponding to the swept scale positions. Amplitudes and phase angles of the two microtransformer output signals (*U*_*ind*1_, *U*_*ind*2_) for two target materials and two operating frequencies (1 and 4 MHz) are presented in Figure 8a. 

The amplitude of the differential signal (*U_diff_* = *U*_*ind*1_ − *U*_*ind*2_) are also shown. Note that the simulated signals’ phases do not exhibit a 90° inductive shift, since the secondary signals originate in line current, which does not have an inductive character. The described inverse operation for the ferromagnetic and the conductive target scale is clearly visible in Figure 8a: magnitudes of displacement-dependent induced voltages |*U*_*ind*1,2_| for the copper scale are always lower due to an increased eddy-current induced energy caused by larger copper conductivity. Furthermore, the maxima and the minima of voltages |*U*_*ind*1,2_| oppose each other for the two materials. At 1 MHz excitation frequency, the ferromagnetic effect manifested in steel overcomes the eddy-current effect in copper, resulting in greater modulation (larger |*U_diff_*|). At 4 MHz, the modulation approximately equalizes, indicating the increasing prominence of the eddy-current effects with rising frequency. As the phase modulation (arg (*U*_*ind*1,2_)) is related to the eddy current losses, its characteristics are not opposite for the two materials. It can be seen how the shape of the differential signal (converted to the time domain in Figure 8b) is much more sinusoidal in comparison to the direct transformer outputs’ amplitudes |*U*_*ind*1,2_|, indicating the importance of the differential operation of such sensors.

The secondary voltages are then subtracted and transformed from the complex domain into a time-domain signal *u_diff_*, shown in Figure 8b. The actual simulated results ranging between 0–1000 µm are duplicated, resulting in 0–2000 µm range, to better illustrate the signals’ shape. For the purpose of the analysis of the demodulation principle, an arbitrary frequency *f_c_* of this signal can be chosen, in this case 1000-times higher than the frequency of the scale movement. During the operation of an actual device, this ratio can be much higher; however the usage of a lower ratio does not affect the illustration of the principle.

#### 2.2.1. Signal Demodulation

The synchronous demodulation method (also: coherent detection) is based on mixing (*i.e.*, multiplying) the modulated signal with a sinusoidal signal of the same carrier frequency *f_c_* and phase ϕ_*mix*_. The resulting signal is composed of an AC signal with 2*f_c_* frequency and a DC component in a linear correlation with the modulation of the signal and the mixing signal phase ϕ_*mix*_ [30] (p. 96).

To suppress the AC component of the signal *U_diff_*, a low-pass filter (LPF) is employed. A fourth-order Butterworth LPF with the 3dB frequency margin of 1% *f_c_* was used, resulting in the demodulated signal *u_diffmix_*. The shape of this signal depends on different material and geometric properties of each scale. The result of the coherent demodulation is also strongly dependent on ϕ_*mix*_, as demonstrated in Figure 8b. Therefore, an optimal mixing signal phase ϕ_*mix*_ can be determined for each target configuration to maximize the output signal amplitude, as will be demonstrated in Section 3 when discussing the microsystem characterization.

To establish the linear position using the arctangent of quadrature signals (Equation (1)), a sinusoidal shape of the position-dependent output signal is required. As its figure of merit, the total harmonic distortion (THD) [31] (p. 83) for the first five harmonics of the demodulated signal has been calculated, which is also stated next to the corresponding phases ϕ_*mix*_ in Figure 8b.

#### 2.2.2. Scale Tilting

**Figure 9:**
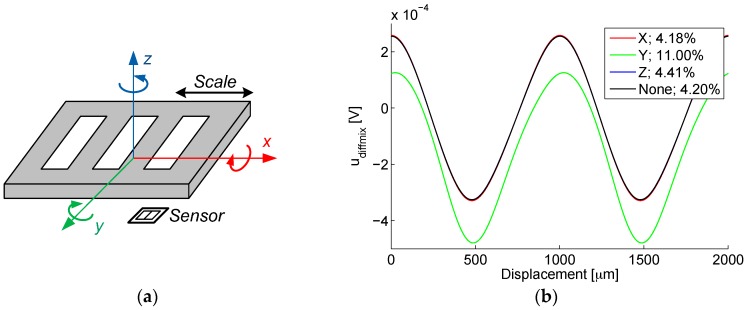
(**a**) The simulated scale rotation (3°) axes shown over the sensor with the microcoils; (**b**) the target rotation simulation results (the demodulated and filtered *u_diffmix_* signal). Signal THDs are given in the legend.

Another issue worth addressing is the tilting of the scale over the sensor, which is inevitable at a sensor operating in an actual application. The tilting was analyzed using the same methodology and the same simulation setup as presented in Section 2.2. The target scale was rotated for an angle of 3° in three dimensions, as depicted in Figure 9a. The simulation was carried out with a copper scale at 4 MHz and ϕ_*mix*_ = 0°; its results are shown in Figure 9b.

The simulation results show that the rotation of the target scale in *x* and *z* axis does not significantly affect the output differential signal *u_diffmix_*, as the results virtually align with the situation without tilting (*None*). However, a notable deviation occurs when the target is tilted longitudinally (*y* axis): each microtransformer in the pair has a different target coupling characteristics due to the different height of the scale over each microtransformer.

Their outputs are not complementary any more, resulting in increased offset and distortion of the differential signal, which is also reflected in the THD increase. For other two (*x* and *z*) axes, the effect of the tilt is almost equal for both microtransformers in a pair, so it is still compensated by the subtraction of their output signals.

### 2.3. ASIC Design and Simulation

**Figure 10:**
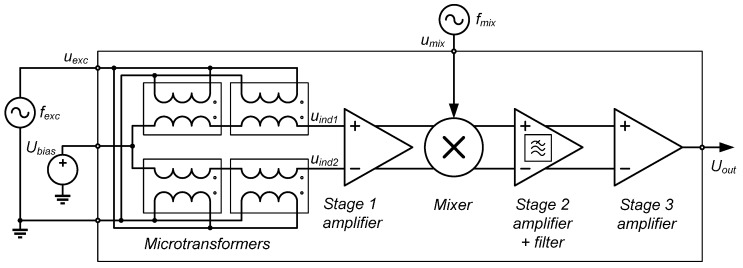
The measurement channel for a single chain of microtransformers, as realized in the prototype ASIC. For the described synchronous demodulation method, *f_mix_* equals *f_exc_*. The DC biasing voltage of the IC *U_bias_* is set to 2.5 V, *i.e.*, to the half of supply voltage (5 V).

We have already introduced the synchronous demodulation method for the differential microtransformer signal. This section describes the actual measurement channel realized in the prototype ASIC, depicted schematically in Figure 10. Cadence Virtuoso Analog Design Environment software (Spectre Simulator) was used as the main tool in the analog front-end design process.

As previously explained, two differential transformers are wired serially to increase the signal level. Fully differential design is used for signal amplification, mixing, and filtering, due to the symmetric nature of the problem (subtracting the signals of two identical microtransformers), improved noise immunity and common mode rejection as well as simpler DC biasing ([32], p. 100–102). The amplification is realized in three stages. Due to different requirements of each particular stage, three different operational amplifiers had to be designed. The first wideband amplifier in the chain employs a telescopic topology due to its required high frequency range ([32], p. 297–299), featuring a simulated DC closed-loop gain of 6 and a gain-bandwidth product of 72 MHz with two serially connected microtransformers at the input. The level of the output signal can be controlled by the amplitude of the mixing signal. A single-balanced Gilbert cell mixer is used ([33], p. 418). The second-stage DC gain is 15. This amplifier employs a simpler Miller topology ([32], p. 296).

The second-stage amplifier also functions as a LPF for the suppression of the excitation frequency *f_exc_*: its 3dB corner frequency is designed to be 325 kHz. This relatively high corner frequency allows for an optional operation of the system with heterodyne mixing ([30], p. 128), if the frequency *f_mix_* is set to a value different from *f_exc_*, resulting in a mixer output signal with a frequency of *f_out_* = |*f_exc_* − *f_mix_*| (another component with a frequency of *f_exc_* + *f_mix_* is suppressed by the LPF). The third-stage amplifier subtracts the signals of the positive and negative signal path, amplifies this difference with a gain of 100, and serves as the output buffer.

**Figure 11:**
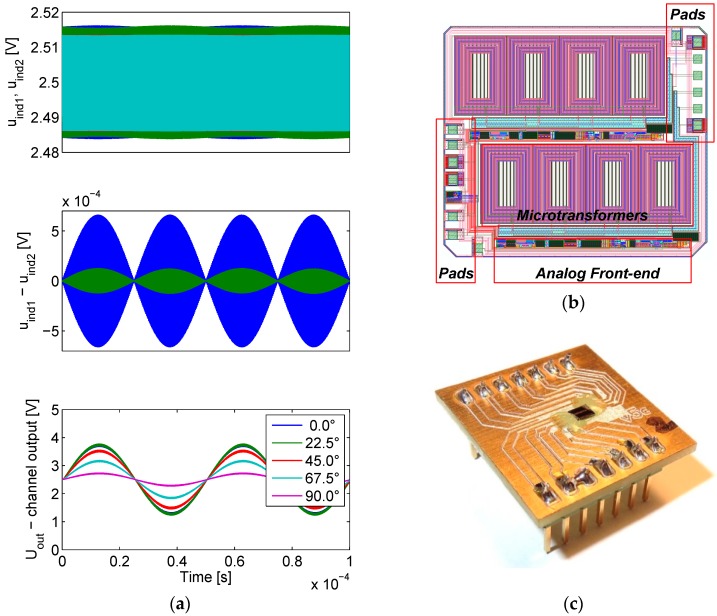
(**a**) Results of Cadence simulations of the measurement channel at 4 MHz operation frequency. In the bottom figure, the mixing signal phase ϕ_*mix*_ is swept between 0°–90°; (**b**) The layout of the integrated circuit, comprising two identical quadrature channels; (**c**) The fabricated ASIC prototype, mounted on a test PCB.

The results of the IC simulation are presented in Figure 11a. The circuit presented in Section 2.1 has been used to model the differential transformers. The scale modulation (with a frequency *f_scale_*) was introduced into the model by sinusoidally varying the value of inductor coupling coefficient *k*, resulting in a symmetric amplitude-only modulation. For this purpose, the model circuit was rewritten into a high-level analog behavioral model description language Verilog-A [34], which allows for the dynamic variation of the model parameters. The modulation was configured to approximate the performance of the FEM-simulated copper scale at 4 MHz when the secondary windings had no load connected. An agreement has been found when the coupling coefficient function was set to:

(3) $$k=0.43+\left(0.43\cdot 0.02\right)\sin\left(2\pi f_{scale}t\right)$$

When the amplifier is connected to the microtransformer, the coil output signals are reduced due to the impedance of the amplifier feedback connected to their output. The effect of this loading is visible in the middle chart in Figure 11a, showing the unloaded differential signal in blue color, and the loaded differential signal in green. The top chart also shows the unloaded (green and blue) and loaded (light green and red) positive and negative coil output signals.

Since the microtransformer and the first-stage amplifier introduce respective 90° and 180° (low-frequency values) phase shifts, with the actual simulated total phase shift being 243° at 4 MHz due to poles in the transfer characteristic, this value is added to the simulated mixing signal phase ϕ_*mix*_, assuring the mixing and demodulated signal coherency at ϕ_*mix*_ = 0°.

The excitation voltage *u_exc_* and mixing voltage *u_mix_* amplitudes are set to the same values used later in the characterization of the IC, *i.e.*, 2.5 V and 0.25 V respectively. The bottom graph in Figure 11a indicates that the amplitude maximum of the output signal *U_out_* occurs when ϕ_*mix*_ = 0°. Since the phase modulation is not modeled, the mixing signal and the signal being demodulated are in-phase at zero ϕ_*mix*_, resulting in maximal amplitude. Such simulations that neglect the phase modulation of the target have been used primarily to adjust the gain of the amplifier chain.

The layout of the microsystem is depicted in Figure 11b. The silicon footprint of the analog front-end is relatively small in comparison to the area of the microtransformers. The fabricated ASIC prototype on a mounting PCB is presented in Figure 11c. The ASIC silicon die dimensions are 2500 by 2200 µm, at a 300 µm wafer thickness. As the bonding wires are thin (several 10 µm), they can easily bend or tear in case of a contact with scale, which is highly possible. Therefore they must be protected. A protective epoxy coating is used for their encapsulation.

## 3. Measurement Results

**Figure 12:**
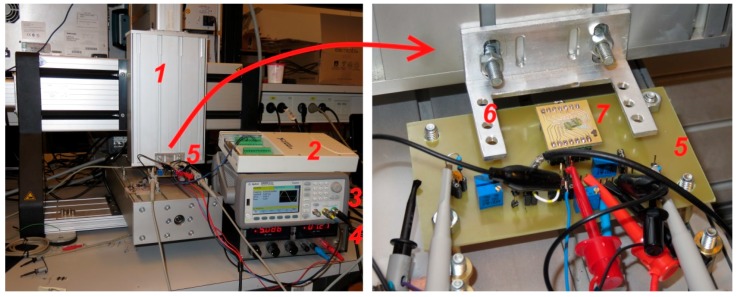
The microsystem characterization setup. 1—three-axis motorized manipulator; 2—16-bit data acquisition interface (DAQ) NI USB-6251; 3—function generator Agilent 33500B; 4—DC power supply; 5—main PCB board, providing the DC bias voltages for the ASIC, and connection terminals; 6—measurement scale support (the scales (not shown, scale types presented in Figure 6) are positioned over the ASIC, supported by the forks); 7—the sensor PCB board (Figure 11c).

Figure 12 presents the laboratory setup for the microsystem characterization. The scale, fixed to a support fork, is moved over the microsystem by a computer-controlled three-axis motorized manipulator. The ASIC excitation and mixing signals are generated by a function generator. The signal is sampled by a DAQ computer interface. A personal computer (not shown) uses MATLAB scripts to control the manipulator, the function generator, the DAQ interface, and to process the acquired signals.

The scale was placed to such vertical position that it barely made contact with the coating surface. The coating is approximately 200–250 µm thick, meaning that the IC-to-scale distance also had such value. Unfortunately the coating thickness could not be accurately determined due to the unevenness of the coating (as it is applied by hand) and the lack of suitable accurate measurement devices. The effect of coating unevenness can be estimated by the scale tilting simulation results provided in Section 2.2.2.

All presented measurements have been carried out with an RC filter at the IC output with a corner frequency of 100 Hz to remove the remaining AC component. Sinusoidal signals with 2.5 V and 250 mV amplitude were used as the excitation and the mixing signal respectively.

### 3.1. Optimal Operating Frequency and Phase

In the first measurement, the frequency of the excitation signal *f_exc_* (equaling the frequency of the mixing signal *f_mix_*) and the phase of the mixing signal ϕ_*mix*_ were swept in the range between 1–4 MHz (0.5 MHz steps) and 0°–90° (10° steps) to determine the optimal operating conditions of the microsystem for both scale types. The excitation signal phase was 0°. The peak-to-peak IC output voltage of the first measurement channel (*U_pp_*_1_) was chosen as a figure of merit.

**Figure 13:**
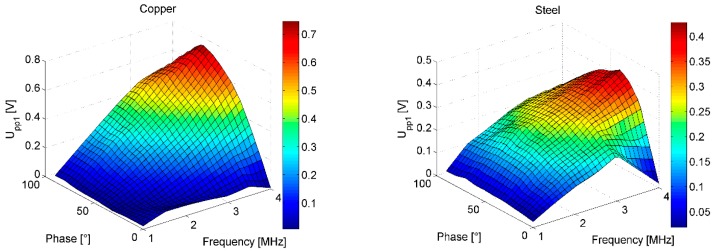
Determining the optimal operating frequency *f_exc_* and the mixing signal phase ϕ_*mix*_ of the microsystem for each of the scales. The color represents the *U_pp_*_1_ voltage. The images are interpolated.

Since the IC output voltage is DC, in this context peak-to-peak voltage means the difference between the maximum and the minimum output voltage over the scale linear displacement range of 2 mm. The positioning step was 50 µm. The results are presented in Figure 13.

**Figure 14:**
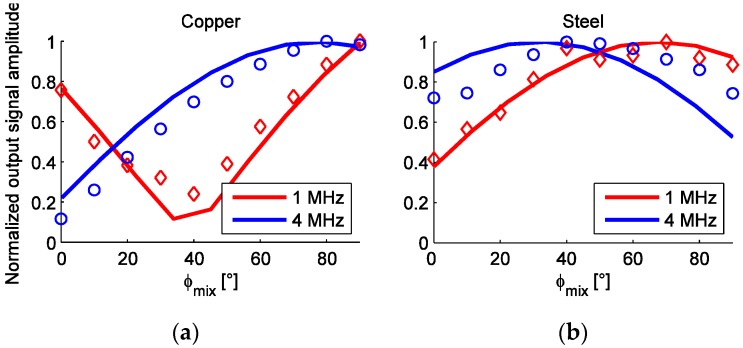
The dependence of ϕ_*mix*_ on the *U_pp_*_1_ microsystem output voltage at 1 and 4 MHz. Lines denote simulated values, while the measured points are marked by circles and diamonds. All amplitude values are normalized. (**a**) Results for copper scale; (**b**) Results for steel scale.

Figure 14 demonstrates the agreement of the simulated (using the method described in Section 2.2 and Figure 8b) and measured dependence of the output signal *U*_*pp*1_ amplitude on ϕ_*mix*_. Similarly as before, the total phase shift of the microtransformers and the first-stage amplifier (263° at 1 MHz, 243° at 4 MHz) needs to be added to the simulated coil output signals to correspond to the measurements.

**Figure 15:**
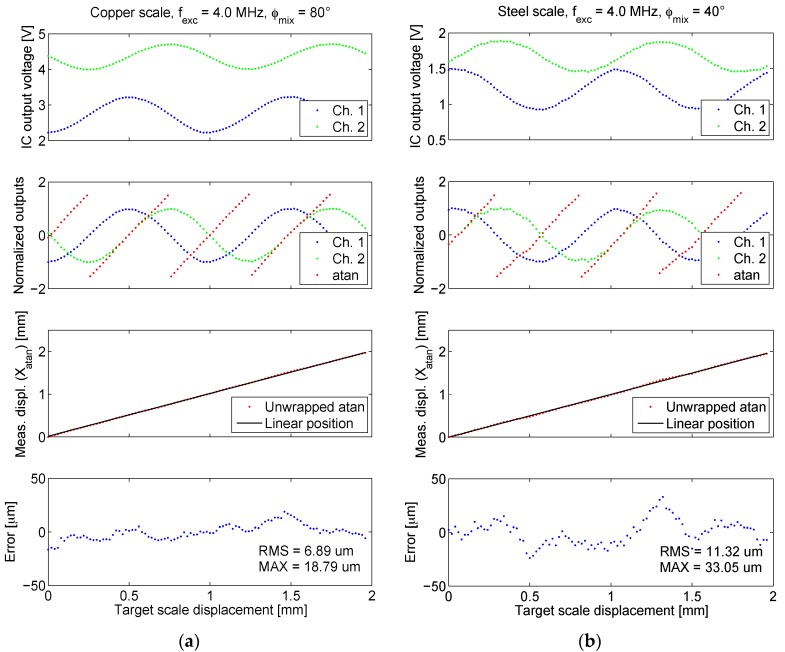
The results of the microsystem linear displacement characterization at optimal excitation frequency and mixing signal phase for each scale type. Results for two scale period lengths are shown. The unwrapped arctangent function for the normalized sensor outputs is also shown, along with errors relative to the reference linear position. (**a**) Results for copper scale; (**b**) Results for steel scale.

Then, the experimentally determined optimal (*i.e.*, providing the maximal output amplitude) frequency and phase for both scales (4 MHz; 80° and 40° for copper and steel scale, respectively), were used to carry out more precise target displacement sweeps with a positioning step of 20 µm, presented in Figure 15. Periodically grated scales (see Figure 6) give periodic quadrature outputs (channels 1 and 2). The starting position was arbitrary for both scales and the measurement is incremental: the displacement is always zero at the starting position.

Since the voltages of both channels are not in the same range, they need to be normalized between 1 and −1 before the calculation of the arctangent of their ratio:

(4) $$\text{arctan}\left(\frac{U_{pp2}}{U_{pp1}}\right)$$

which should ideally be periodically linear over a half of the scale period (*P*/2=0.5 mm). After scaling and unwrapping, *i.e.*, removing jumps, the arctangent should present a linear function *X_atan_(x)* of the displacement *x* [25], as shown in Figure 15. In the bottom chart, the nonlinearity error *E* of the arctangent function is shown, defined as the difference of the measured displacement *X_atan_* and the reference linear position *X_ref_*:

(5) $$E\left(x\right)=X_{atan}\left(x\right)-X_{ref}\left(x\right)$$

Commonly, analog quadrature signals (*U_pp_*_1_ and *U_pp_*_2_ in this case) of any incremental encoder type are driven into an interpolator device (typically also an ASIC) [35,36]. To return the discrete position information, interpolators employ advanced mixed signal processing methods for amplitude (e.g., automated/programmable gain control), offset, and phase correction of the signal nonidealities, which can be for example caused by scale tilting. As the presented microsystem is already fabricated as an ASIC, there also exists a potential for a same-chip implementation of an interpolator, resulting in further improved cost-efficiency and functionality.

The sensitivity of the discussed microsystem, defined (Equation 6) as the ratio of the output voltage change over a period Δ*U_pp_* = max(*U_pp_*) − min(*U_pp_*) and the scale period *P*:

(6) $$S=\frac{\Delta U_{pp}}{P}\left[\frac{V}{\text{mm}}\right]$$

depends on many variables, namely the target material, shape, vertical displacement, as well as the ASIC input signals’ parameters: shape, amplitude and frequency of the excitation and the mixing signal, and also the phase shift between the excitation and mixing signal. Sensitivities for the optimal setup with the parameters as given in this section (results in Figure 15), are presented in Table 2. Maximal and RMS values of nonlinearity error *E* (Equation (5)) are also given for both scales.

### 3.2. Target Vertical Displacement

**Figure 16:**
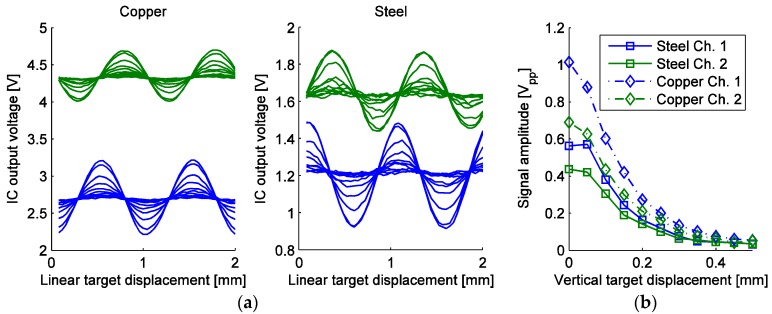
The results of the microsystem vertical target displacement sweep for both scale types; (**a**) the measured IC output voltages for both channels at all vertical target positions; (**b**) The relation of the measured V_pp_ voltages from (**a**) to the vertical target displacement. The linear starting position was arbitrary for both scales.

The same optimal frequency and phase have also been used for the measurement of the dependence between the target vertical displacement and output signal *U_pp_* amplitude in a scale period, given in Figure 16. The characterization was carried out in the vertical displacement range between 0 and 0.5 mm in 50 µm steps. The horizontal step was also 50 µm. The vertical displacement is measured from the top of the IC epoxy coating with the approximate thickness of 200–250 µm (see Figure 11b). To establish the effective vertical displacement between the top surface of the IC and the target, the measured vertical displacement displayed in Figure 16c must be added to the coating thickness. It can be seen that the vertical displacement of the target has a significant effect on the target sensitivity, reducing it for an approximate factor of three when the target is raised for 200 µm. As the arctangent method is used for linear position determination, only the ratio of the quadrature signals is important, which remains constant regardless of vertical displacement once the quadrature channels are normalized (commonly done in the interpolator). The usable vertical range of operation is limited by the required signal-to-noise ratio of the given application.

For a microtransformer comprising concentric coils covered with a target, monotonic diminishing of the target coupling effect magnitude on its secondary voltage is expected if the target is being vertically displaced. This is confirmed by the measurement data in Figure 16b with the exception of a visible flatness of the characteristic curves at low vertical displacements at the steel scale. This can be explained by the flexibility of the steel scale due to its thinness (0.35 mm). The contact point between the sensor and the target might have been set slightly too low, causing upwards bending of the scale in its center, therefore raising it marginally above the IC coating surface, with the bend leveling in the next height step, placing the scale closer to the IC. Due to its rigidness, this did not occur with the thicker copper scale. Another possible explanation is provided by Tseng and Xie [18], who have studied a conductive micromirror plate placed over a transmitting and a receiving microcoil positioned in line. The maximum effect of the plate on the inter-coil inductive coupling occurred at a certain vertical distance between the coils and the plate. It is possible that a similar effect also manifests here between primary and secondary windings of different microtransformers, resulting in distorted characteristics at small vertical displacements.

## 4. Discussion

Simulation and measurement results of an inductive linear displacement sensing microsystem have been presented. The main purpose of the simulations has been the dimensioning of the gain of the analog front-end circuits and the prediction of the output signal shape, which proved successful. The output signals’ mean value should be close to *U_bias_* = 2.5 V according to theory and simulations. We attribute the exhibited DC offset to the asymmetries in the positive and negative signal path, caused by on-chip element mismatch and fabrication tolerances, which manifest strongly due to the high gain of the system. The differences in the offset voltages and sensitivities between the channels are also significant. They can be attributed to the mispositioning of the target scale (e.g., its tilting relatively to the ASIC, as suggested by the simulation results in Section 2.2.2.). This could be caused by the unevenness of the top coating. Within-die CMOS process parameter variations [32] due to the large distance between the front-end electronics for each channel are another possible reason. In future ASIC improvements, the described offsets and differences can be alleviated by implementing trimming structures into the analog front-end. Ideas for offset compensation and transimpedance amplifier utilization to improve the performance of similar sensor designs are also suggested in [22].

With the microsystem input stimulation equalized between Cadence simulations and the measurement setup, the output signal amplitudes have been comparable. A measurement able to assess a more exact agreement between the simulated and measured output voltages is demanding to design from a mechanical point of view, since the IC-to-scale distance and parallelism would have to be very precisely controlled, as indicated by the strong dependence between the vertical displacement of the target and IC output voltage (Figure 16c). The FEM simulation results have predicted approximately equal sensitivity of the signal for the steel and copper scale at 4 MHz. This does not agree with measurements, which display significantly better sensitivity for the copper scale. We attribute this difference to the imprecise material properties of steel used in the FEM simulation due to lack of material data. The measured inferior performance of the steel scale agrees with literature [17].

The simulations have predicted smaller distortion for the steel scale which should reflect in better linearity. This does not agree with measurements displaying a greater nonlinearity error for the steel scale (11.32 µm RMS *vs.* 6.89 µm RMS for copper). Beside lower signal levels due to the lower sensitivity, the reason for the inferior linearity of the steel scale may also be in its imprecise manufacturing due to laser cutting tolerances (beam width 40 µm), possibly making the scale half-periods asymmetric.

The system exhibits a resolution of at least 20 µm using both transformer steel and copper target scales, as indicated by the monotonicity of arctangent waveforms (Figure 15). We were unable to assess the performance at smaller displacement steps due to the step resolution limitation of mechanical manipulator used for the characterization.

However, the simulated and the measured results for the optimal mixing phase angle ϕ_*mix*_ are in good agreement for both materials, except for the steel scale at 4 MHz, which exhibits a 10°–20° deviation of the amplitude maximum. This can again be attributed to the inexact steel material properties used in the simulation.

### Theoretical Resolution Limit

The theoretical resolution limit of the discussed microsystem is set by the thermal noise of the sensor (*i.e.*, the secondary microtransformer winding) and the desired bandwidth, dictated by the maximum target movement speed. If, for example, the target having a period of 1 mm moves with 1 m/s, an analog front-end bandwidth (after mixer) of 1 kHz is required. As the secondary resistance of a single microtransformer is approximately 4 kΩ, totaling in 16 kΩ of sensor output resistance (four coils per channel are used), this returns 0.51 µV_rms_ thermal input noise at 20 °C [32] (p. 210). At 4 MHz excitation frequency, the simulated input sensitivity (directly at the microtransformer output) at copper target (at 1 mA primary current; Figure 8b) is in the range of 1 mV/mm. Therefore, the resolution limit in the described situation is 0.51 µm at 1 kHz bandwidth (1.6 µm at 10 kHz). Lower movement speed specifications, allowing for stronger low-pass filtering, would reduce the required bandwidth, resulting in an improvement of the noise performance. The resolution can also be improved by raising the excitation signal frequency or magnitude.

## 5. Conclusions

### 5.1. Summary

We have presented the operational background and the design flow for a monolithic linear displacement measurement microsystem based on integrated microtransformers and synchronous demodulation. The system is intended to be used in a linear incremental encoder.

In the design process, we have relied on FEM modeling, parasitic extraction, system-level mathematical and behavioral modeling, as well as CMOS circuit-level simulation of the microsystem. A prototype ASIC, fabricated using a low-cost 4-metal conventional 350 nm CMOS process, was described, followed by the prototype characterization. A considerable agreement between the simulation results and the measurements was demonstrated, confirming the appropriateness of the presented design methodology for the use in the design process of miniaturized inductive sensor systems.

The key novelty and advantage of the prototyped microsystem is its monolithic integration; sensor elements and analog front-end are jointly fabricated on a single silicon die using a non-modified cost-efficient CMOS process. This assures ease and repeatability of the fabrication of such microsystems, as well as their easy implementation in target encoder applications. By employing inductive technology, the need for an external light or magnetic field source as required by conventional optical or magnetic position encoders, is eliminated.

The prototype demonstrates a resolution of at least 20 µm using both steel and copper measurement scales. The best performance (sensitivity of 0.99 V/mm and RMS nonlinearity error of 6.89 µm) was achieved at 4 MHz operating frequency with a scale fabricated as a conventional PCB with 35 µm copper plating. The system directly outputs two standard DC quadrature outputs, which can be interpolated using a standard arctangent method to obtain a linear position signal.

### 5.2. Outlook

To the best of our knowledge, there are no other comprehensive reports of such devices (integrated incremental displacement measurement system operating with microtransformers thoroughly fabricated in CMOS technology), in the available literature, so a direct comparison is difficult to make. This work should therefore be regarded as a proof of concept and feasibility of such device, as well as a presentation of suitable design and modeling methodology.

Based on reports presenting many small scale, yet non-integrated eddy-current displacement sensors with submicrometer resolutions (e.g., [18]—20 nm, [17]—0.5 nm, [9]—120 nm), we believe that with further improvements focusing on the processing electronics, such integrated design will allow to approach its resolution limit of few µm, set by the resistance of the microtransformer secondary winding and the desired movement speed. This will make the system comparable to conventional inductive [37] and magnetic incremental encoders [38], both types reaching resolutions close to 1 µm. As the system is able to operate with non-ferromagnetic scales, it does not suffer from hysteresis, which presents another disadvantage of magnetic systems [5,38].

The main goal of the proposed system is not to achieve extreme measurement resolutions provided by optical encoders (down to 1 nm [8]), but to find its use in applications requiring high cost efficiency, which is enabled by monolithic CMOS fabrication. The presented work can also serve as a basis and encouragement for further research in the field. We plan to continue our work on integrated inductive microsystems, in which the design methodology introduced in this article will be of key importance.

## Figures and Tables

**Table 1:** The components’ values for the model circuit presented in Figure 3c.

Components	*R*_1_, *R*_2_	*R*_3_, *R*_4_	*L*_1_, *L*_2_	*L*_3_, *L*_4_	*C*_1_	*C*_2_	*C*_3_	*k*_1_, *k*_2_
**Value**	2657 Ω	1816 Ω	1.16 µH	658 nH	3.55 pF	3.4 fF	2.39 pF	0.429

**Table 2:** The comparison of the sensitivities *S* and errors *E* for the both targets for results given in Figure 15.

Target	Copper	Steel
***S* (Ch. 1)** $\left[\frac{V}{\text{mm}}\right]$	0.99	0.57
***S* (Ch. 2)** $\left[\frac{V}{\text{mm}}\right]$	0.71	0.44
**max *(E)*** [μm]	18.79	33.05
**rms *(E)*** [μm]	6.89	11.32

## Abbreviations

FEM: Finite Element Method
IC: Integrated Circuit
CMOS: Complementary Metal Oxide Semiconductor
ASIC: Application Specific Integrated Circuit
ESD: Electrostatic Discharge
PCB: Printed Circuit Board
THD: Total Harmonic Distortion
GDS: Graphic Database System (an industry standard file format for IC layout database exchange)
LPF: Low-Pass Filter
RMS: Root Mean Square
